# Supplementary figures and images for: Epithelial Infection With Candida albicans Elicits a Multi-System Response in Planarians
Source: Front Microbiol. 2021 Jan 14;11:629526. doi: 10.3389/fmicb.2020.629526 (PMC7840899; doi:10.3389/fmicb.2020.629526)

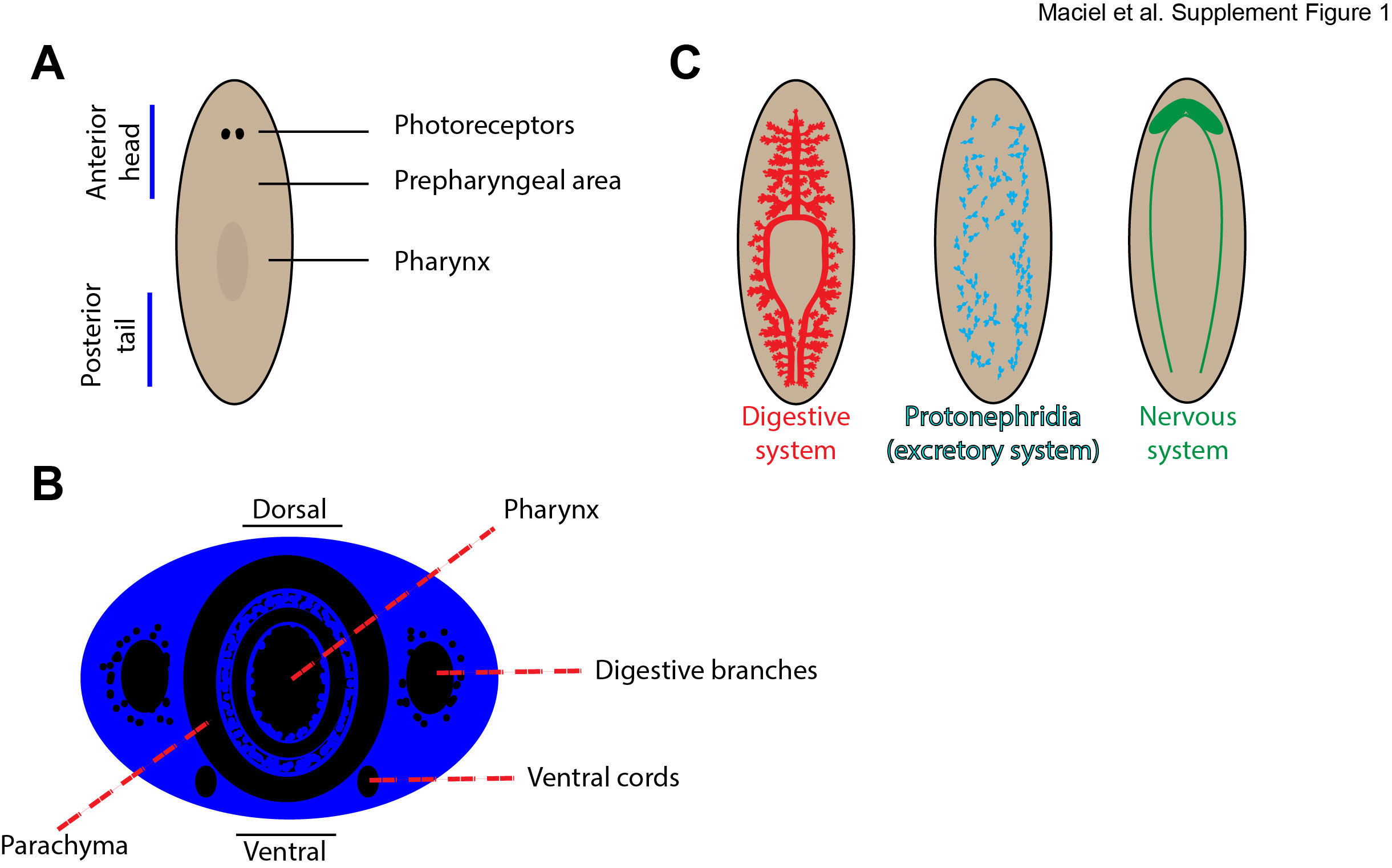

Supplement: Supplementary Figure 1 — Orientation and basic anatomy of the planarian body. (A) Demonstrates the orientation the planarians in the whole-mount images. (B) Depiction of a transverse cross section of a planarian in the orientation found in the images throughout the figures. (C) Depiction of the digestive system, nervous system, and protonephridia of planarians. [file Image_1.JPEG]

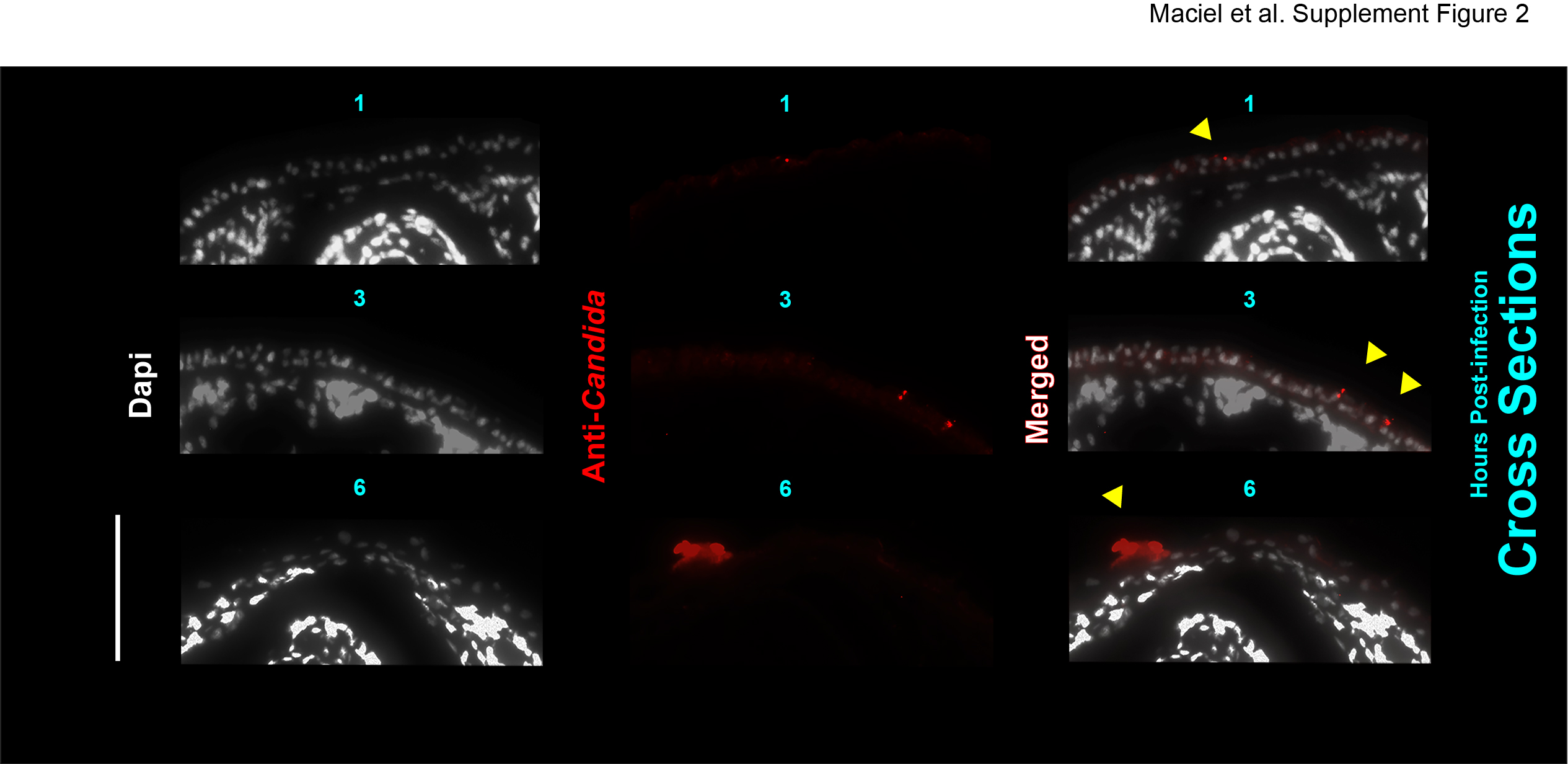

Supplement: Supplementary Figure 2 — Cross section images of the planarian body during early infection with C. albicans. Individual transverse cross section images are displayed from the dorsal side of the planarian body showing staining with the DAPI nuclear marker (white signal), anti-Candida antibody (red signal), and merged images, at 1, 3, and 6 h post-infection. Yellow triangles indicate notable C. albicans aggregates. [file Image_2.JPEG]

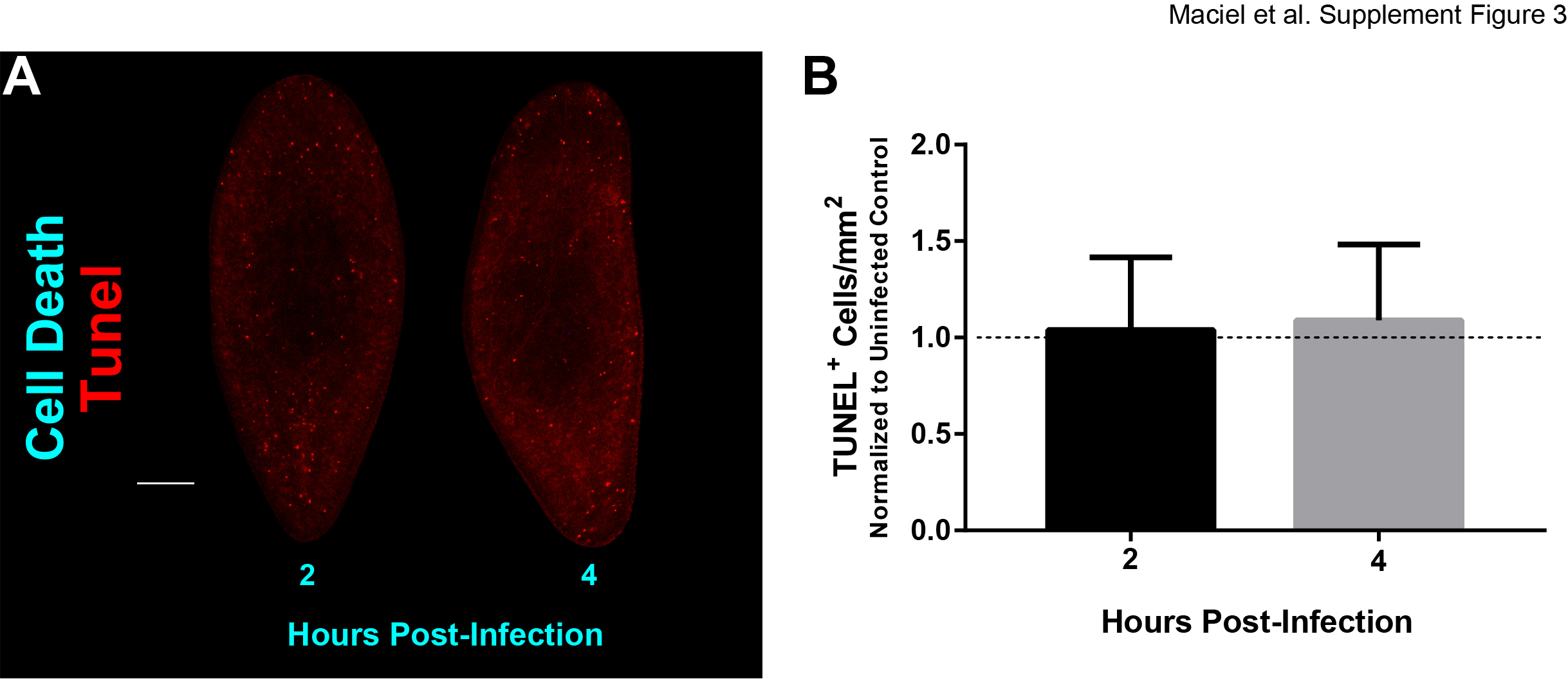

Supplement: Supplementary Figure 3 — Cell death occurring during the early stages of infection with C. albicans. (A) TUNEL straining (red foci) was performed in uninfected planarians to compare animals infected at 2 and 4 h post-infection using 25 million cells/mL of C. albicans. (B) Levels of TUNEL+ cells in the planarian tissue 2 and 4 h post-infection normalized to an uninfected control. Cell death experiments consisted of two biological replicates using four animals each. All graphs represent mean ± SEM. Scale bar is 200 μm. [file Image_3.JPEG]
